# Aducanumab, gantenerumab, BAN2401, and ALZ-801—the first wave of amyloid-targeting drugs for Alzheimer’s disease with potential for near term approval

**DOI:** 10.1186/s13195-020-00663-w

**Published:** 2020-08-12

**Authors:** Martin Tolar, Susan Abushakra, John A. Hey, Anton Porsteinsson, Marwan Sabbagh

**Affiliations:** 1 Alzheon, Inc., 111 Speen Street, Suite 306, Framingham, MA 01701 USA; 2 University of Rochester School of Medicine and Dentistry, Rochester, NY USA; 3 Cleveland Clinic Lou Ruvo Center for Brain Health, Las Vegas, NV USA

**Keywords:** Alzheimer’s disease, Beta amyloid oligomers, Anti-oligomer agents, Anti-amyloid antibodies, APOE4 genotype, Aducanumab, Gantenerumab, BAN2401, ALZ-801

## Abstract

The body of evidence suggesting a causative, initiating role of beta amyloid (Aβ) in the pathogenesis of Alzheimer’s disease (AD) is substantial. Yet, only a few anti-amyloid agents have shown meaningful efficacy in clinical trials. We evaluated the unifying characteristics of anti-amyloid agents with positive clinical or biomarker effects in long-duration trials and analyzed how pharmacological characteristics determine their clinical product profiles. Four agents with the potential for near term approval fulfill these criteria: the injectable antibodies, aducanumab, gantenerumab, and BAN2401, and a small molecule oral agent, ALZ-801. Aducanumab and BAN2401 showed significant efficacy on both clinical and biomarker outcomes; gantenerumab showed significant biomarker effects, with no clinical efficacy reported to date; and ALZ-801 showed significant clinical effects in the high-risk population of patients homozygous for the ε4 allele of apolipoprotein E gene (APOE4) and a dose-dependent preservation of hippocampal volume. We explored how the pharmacological properties of these agents, namely selectivity for Aβ oligomers, plasma half-life, brain penetration, and time to peak brain exposure, determine their clinical profiles. A crucial characteristic shared by these agents is their ability to engage neurotoxic soluble Aβ oligomers, albeit to various degrees. Aducanumab and gantenerumab partially target oligomers, while mostly clearing insoluble amyloid plaques; BAN2401 preferentially targets soluble protofibrils (large oligomers) over plaques; and ALZ-801 blocks the formation of oligomers without binding to plaques. The degree of selectivity for Aβ oligomers and brain exposure drive the magnitude and onset of clinical efficacy, while the clearance of plaques is associated with vasogenic brain edema. Only the highest doses of aducanumab and BAN2401 show modest efficacy, and higher dosing is limited by increased risk of vasogenic edema, especially in APOE4 carriers. These limitations can be avoided, and efficacy improved by small molecule agents that selectively inhibit the formation or block the toxicity of Aβ oligomers without clearing amyloid plaques. The most advanced selective anti-oligomer agent is ALZ-801, an optimized oral prodrug of tramiprosate, which demonstrated efficacy in homozygous APOE4/4 AD subjects. ALZ-801 selectively and fully inhibits the formation of Aβ42 oligomers at the clinical dose, without evidence of vasogenic edema, and will be evaluated in a phase 3 trial in homozygous APOE4/4 patients with early AD. In addition to clinical measures, the phase 3 trial will include cerebrospinal fluid, plasma, and imaging biomarkers to gain further insights into the role of soluble Aβ oligomers in the pathogenesis of AD and their impact on disease progression.

## Introduction

The central and early role of beta amyloid (Aβ) in the pathogenesis of Alzheimer’s disease (AD) is supported by numerous genetic, biomarker, and genome-wide association studies in both familial (early-onset) and sporadic (late-onset) AD [1]. The brain amyloid species consistently shown to be associated with acute neuronal toxicity and neurodegeneration in AD are the soluble amyloid oligomers, formed by the aggregation of misfolded Aβ monomers [1–3]. Several preclinical and clinical studies have shown that soluble Aβ oligomers, rather than insoluble aggregates that form plaques and fibrils, are the key amyloid species that initiate neurotoxicity and disease progression in AD [4–8]. Further evidence for the upstream role of Aβ in driving tau pathology and cognitive decline in AD patients comes from recent longitudinal amyloid and tau positron emission tomography (PET) imaging studies. These studies show that cortical amyloid burden must reach a critical threshold before tau pathology spreads from the medial temporal lobes to the neocortex, expediting cognitive decline [9]. This sequence of pathologies implies that amyloid targeted agents should reduce downstream tau pathology and cognitive decline, which is indeed what has been observed in recent clinical trials.

Despite the substantial body of evidence supporting the toxic role of amyloid, only a few anti-amyloid agents have shown significant cognitive benefits in AD clinical trials. A systematic review of the positive and negative trials can provide insights into the critical factors underlying clinical efficacy and accelerate the future development of disease-modifying treatments for AD. In this analysis, we evaluated the mechanisms of action (MOA), selectivity for amyloid species, and pharmacological profiles of late-stage anti-amyloid agents in active development, and correlated these features with clinical and biomarker effects consistent with disease modification in long-duration trials. We also analyzed the benefit-risk profiles of these agents, which may become AD treatments in the near future, in the overall AD population as well as the subgroup of high-risk carriers of the ε4 allele of apolipoprotein E gene (APOE4).

## Methods

This analysis focused on anti-amyloid agents that fulfilled the following criteria: (1) completed phase 3 or phase 2 trials in symptomatic AD patients; (2) demonstrated acceptable safety with treatment duration ≥ 12 months, the regulatory standard for long-term safety; (3) reported significant effects on clinical outcomes or on AD imaging or fluid biomarkers; (4) published mechanisms of action (MOA) and pharmacokinetic (PK) profiles; and (5) in active clinical development. Details of clinical trial designs were obtained from publications or listings on https://clinicaltrials.gov. The imaging biomarkers included volumetric magnetic resonance imaging (MRI) measures and PET imaging studies of amyloid and tau pathologies. The fluid biomarkers in these trials included plasma or cerebrospinal fluid (CSF) assays of amyloid and tau pathology and of downstream neuronal injury. The data on pharmacokinetic characteristics, efficacy, safety, and biomarker effects were tabulated to allow the analysis of benefit-risk profiles.

## Results

Our analysis identified four agents that fulfilled prespecified selection criteria: the anti-amyloid antibodies aducanumab and BAN2401 administered by intravenous (IV) infusions, gantenerumab administered by subcutaneous (SC) injections, and a small molecule oral agent ALZ-801. Anti-amyloid agents that did not fulfill these criteria include antibodies bapineuzumab, solanezumab, and crenezumab that failed to show efficacy in phase 3 trials, and several oral gamma and beta secretase inhibitors that failed to show efficacy and were burdened by unacceptable toxicities in phase 3 trials. Phase 2 agents that did not fulfill these criteria include several active Aβ vaccines that failed to show efficacy, anti-amyloid antibody donanemab, and oral anti-oligomer agents PQ912 and CT1812 that lack both efficacy and long-term safety data.

The four agents that fulfilled all our criteria, aducanumab, BAN2401, gantenerumab, and ALZ-801, share a common feature, namely their interaction with neurotoxic soluble Aβ oligomers, based on published MOA and binding affinity studies. The three antibodies showed different levels of selectivity for soluble Aβ oligomers versus insoluble plaques and fibrils. The fourth agent, ALZ-801, is an oral prodrug of tramiprosate that inhibits the formation of amyloid oligomers without plaque interaction.

### Differential selectivity for Aβ oligomers

Aducanumab, gantenerumab, and BAN2401 are human or humanized monoclonal antibodies that bind with high affinity to aggregated Aβ and promote its removal by Fc receptor-mediated phagocytosis, while showing much lower affinity to monomers [10–14]. However, these antibodies differ in their selectivity to soluble Aβ oligomers versus plaques or fibrils. A direct comparison study of the selectivity of aducanumab and BAN2401 for soluble amyloid oligomers versus insoluble plaques and fibrils reported a 10-fold higher selectivity of BAN2401 for oligomers [10, 11]. Gantenerumab MOA studies showed partial interaction with oligomers [12]; however, direct comparisons with the other two antibodies have not been reported. ALZ-801 selectively inhibits the misfolding of Aβ42 monomers and oligomer formation in an in vitro assay [15–17] and can fully block the formation of amyloid oligomers in the brain at the target clinical dose [16]. ALZ-801/tramiprosate did not bind plaque in a transgenic model of AD [18]. The differential selectivity for soluble Aβ oligomers, clinical and biomarker efficacy data, and safety profiles of the late-stage anti-amyloid agents are summarized in Tables 1 and 2.

**Table 1:** Late-stage anti-amyloid agents: selectivity for amyloid oligomers and clinical and biomarker effects in phase 2 and 3 studies

Clinical and biomarker profile	*Biogen* Aducanumab IV infusion 10 mg/kg monthly	*Roche* Gantenerumab SC injection monthly (225 mg and 1200 mg doses)	*Eisai* BAN2401 IV infusion 10 mg/kg twice per month		*Alzheon* ALZ-801/tramiprosate Oral tablet 265 mg twice daily
**Amyloid oligomer selectivity**	+/−	+/−	++		+++ Blocks formation of oligomers
**Study population**	Early AD All genotypes	Early AD All genotypes	Early AD All genotypes	Early AD APOE4 carriers	Mild AD APOE4/4 homozygotes
**Cognition** ADAS-cog (% benefit versus placebo)	27% *p* = 0.0097	No effect	47% *p* = 0.017	84% Not reported	125% *p* = 0.0001
**Function** CDR-SB (% benefit versus placebo)	22% *p* = 0.012	No effect	26% *p* = NS	60% Not reported	81% *p* = 0.0197
**CSF p-tau** (% benefit versus placebo)	15%	*31%	13%		Not evaluated
**Effects on other biomarkers**	Not reported	*CSF t-tau **CSF t-tau **CSF NfL	CSF neurogranin, CSF NfL		Preservation of hippocampal volume
**Amyloid plaque removal**	+++	+++	+++		No plaque interaction
**Brain edema ARIA-E** (% of overall population and APOE4 carriers)	35% (all genotypes) 42% (APOE4)	^$^28%–^#^42% (all genotypes)	10% (all genotypes)	15% (APOE4)	0% (all genotypes) 0% (APOE4)

*Abbreviations*: *IV* intravenous, *SC* subcutaneous, *NS* not significant, *ADAS-cog* Alzheimer’s Disease Assessment Scale—cognitive subscale, *CDR-SB* Clinical Dementia Rating—Sum of Boxes, *ARIA-E* amyloid-related imaging abnormalities with effusion or edema

Assessment of amyloid oligomer selectivity: relative binding activity for soluble oligomers and protofibrils was measured by Biacore surface plasmon resonance. BAN2401 showed differential binding (*K*_D_) at 1.32 nM versus aducanumab 138 nM [10]; gantenerumab displays comparable affinity for oligomers and fibrils, and about 10× lower affinity for monomers [12]; ALZ-801/tramiprosate fully inhibits the formation of oligomer in the brain at target clinical dose [15]

Data sources: aducanumab phase 3 studies [11, 19]; gantenerumab phase 3 studies at 225 mg SC and *DIAN-TU at mix of 225 mg SC and 1200 mg SC [13, 20, 21]; gantenerumab safety data: **phase 3 SCarlet RoAD study [13]; ^$^Marguerite RoAD open-label extension study [22]; ^#^amyloid PET open-label extension study [20]; BAN2401 phase 2 study [23]; ALZ-801: tramiprosate phase 3 study [15–18, 24]

**Table 2:** Anti-amyloid antibodies in early AD trials: clinical efficacy, safety, and PET imaging effects

Antibody	Antibody type	Target amyloid species	Clinical efficacy	PET imaging	Safety profile
**Aducanumab** *Biogen*	IgG1 human N-terminal	Plaque, fibrils > > oligomers	ADAS-cog 27% CDR-SB 22%	Amyloid PET ~ 80% Tau PET: significant reduction	ARIA-E 35% ARIA-H 18% Headache 20%
**Gantenerumab** *Roche*	IgG1 human N-terminal and central	Plaque, fibrils, and oligomers	ADAS-cog: no effect to date, phase 3 ongoing CDR-SB: no effect to date, phase 3 ongoing	Amyloid PET ~ 75% Tau PET: not evaluated	ARIA-E 28–42% ARIA-H 15–25% Injection site erythema 13%
**BAN2401** *Eisai*	IgG1 humanized N-terminal	Large oligomers (protofibrils) > plaque	ADAS-cog 47% CDR-SB 26%	Amyloid PET ~ 90% Tau PET: not evaluated	ARIA-E 10% ARIA-H: not reported Infusion reactions leading to discontinuations 2.5%

*Abbreviations*: *IgG* immunoglobulin G, *PET* positron emission tomography, *ARIA-E* amyloid-related imaging abnormalities with effusion or edema, *ARIA-H* amyloid-related imaging abnormalities with hemosiderin deposits

Data sources for target amyloid species: aducanumab [10, 11]; gantenerumab [12, 13, 20]; BAN2401 [10, 14]. Data sources for clinical and imaging data: aducanumab [11, 19]; gantenerumab [13, 20–22]; BAN2401 [23, 25]

### Clinical and biomarker efficacy data

Aducanumab showed significant efficacy in the reanalysis of the EMERGE phase 3 trial in early AD, while the ENGAGE phase 3 trial was negative [19]. The highest dose of 10 mg/kg monthly IV infusions showed significant effects on the primary outcome, the cognitive and functional composite measure Clinical Dementia Rating—Sum of Boxes (CDR-SB), and on the cognitive endpoint Alzheimer’s Disease Assessment Scale—cognitive subscale (ADAS-cog). While both phase 3 trials were stopped for futility after an interim analysis, the positive final analysis in the EMERGE trial was supported by significant biomarker effects on phosphorylated tau (p-tau) in CSF and on tau PET imaging [19].

BAN2401, in a large phase 2 trial in early AD, showed significant efficacy at the highest dose, 10 mg/kg IV infusion twice per month [23, 25]. BAN2401 showed significant and clinically meaningful effects on the primary outcome, the Alzheimer’s Disease Composite Score (ADCOMS) [26] and the ADAS-cog, which were larger in APOE4 carriers. BAN2401 also showed significant effects on CSF p-tau and on downstream markers of neuronal injury (neurofilament light chain, NfL) and synaptic integrity (neurogranin) [23, 25].

Gantenerumab, in phase 3 studies in prodromal and early AD, showed no clinical efficacy at 225 mg and 1200 mg doses administered monthly by subcutaneous (SC) injection, while significant effects on CSF p-tau and total tau (t-tau, marker of neuronal injury) were reported [13, 20, 22]. A recently completed study in familial AD, led by the Dominantly Inherited Alzheimer Network Trials Unit (DIAN-TU), enrolled approximately 50 subjects into each of placebo, solanezumab, and gantenerumab arms for 4 years of treatment [21, 27]. This study used the higher dose of 1200 mg monthly SC injection of gantenerumab that has shown acceptable long-term safety [20]. At this dose, gantenerumab showed significant effects on CSF p-tau, t-tau, and NfL at 4 years in the familial AD population, but no clinical efficacy. The antibody solanezumab which selectively binds to Aβ monomers and has minimal brain penetration [28] showed no clinical or biomarker effects in the DIAN-TU study, in line with the negative data from the previous phase 3 trials [29].

ALZ-801 is an optimized prodrug of tramiprosate, which consists of tramiprosate conjugated to the essential amino acid valine, providing improved gastrointestinal tolerability and more consistent plasma levels that lead to increased brain penetration [30]. Tramiprosate, the active agent in ALZ-801, was evaluated in a phase 3 study of mild to moderate AD that enrolled all APOE genotypes and did not show efficacy in the overall study population. Subgroup analyses showed clinical efficacy in the high-risk APOE4 carriers [24]. At the high dose, tramiprosate showed significant efficacy on both the ADAS-cog and CDR-SB co-primary outcomes in homozygous APOE4/4 subjects with mild AD [31]. In addition, a dose-dependent preservation of hippocampal volume was observed in the MRI sub-study [32], further supporting a disease-modifying effect of ALZ-801/tramiprosate treatment.

### Safety results

The clearance of aggregated amyloid from brain vessels is associated with amyloid-related imaging abnormalities with edema (ARIA-E) or microhemorrhage (ARIA-H) and was first reported in trials with anti-amyloid antibodies [33]. The incidence of these events closely correlates with the clearance of cerebral amyloid on PET imaging and is summarized in Tables 1 and 2. At the doses used in phase 3 trials, aducanumab and gantenerumab reported ARIA-E rates of 30% or greater across all genotypes [19, 20], while BAN2401 showed a lower rate of 10% [23], consistent with the lower affinity of BAN2401 for amyloid plaques [10]. The ARIA-E rates were higher in APOE4 carriers, 42% with aducanumab and 15% with BAN2401 [19, 23]. Treatment with ALZ-801/tramiprosate did not cause any ARIA-E events in 426 AD subjects followed by serial MRIs, even in APOE4 carriers [31], suggesting a lack of interaction with amyloid plaque and vascular amyloid.

### Pharmacokinetic properties

The pharmacokinetic (PK) properties, their impact on target engagement of Aβ oligomers, and their translation into clinical benefit were analyzed for the four late-stage anti-amyloid agents. In addition to selectivity for neurotoxic soluble Aβ oligomers, an essential feature of an amyloid-targeted drug is its ability to cross the blood-brain barrier and to achieve sustained therapeutic brain concentrations at levels sufficient to persistently remove oligomers or inhibit their formation, even at trough levels between doses. The time to peak brain levels of a drug is approximately 5 times its plasma half-life (*t*_1/2_) or 5 times the dosing interval, if the dosing interval is longer than the *t*_1/2_. The key features of each product, including dose, route of administration, dosing regimen, and PK profile [11, 14, 30, 34], are summarized in Table 3.

**Table 3:** Late-stage anti-amyloid agents: phase 3 dosing regimens and pharmacokinetic profiles

Agent	Dose per visit and total dose*	Route of administration, frequency, and dosing facility	Plasma half-life	Brain penetration	Time to peak brain steady state exposure**
Aducanumab *Biogen*	10 mg/kg IV 600–750 mg per month	IV infusion, monthly Titration over 6 months Infusion center	21 days	< 1.5%	~ 5 months
Gantenerumab *Roche*	1200 mg SC 1200 mg per month	SC injection, monthly Titration over 6 to 10 months Clinic	22 days	~ 1%	~ 5 months
BAN2401 *Eisai*	10 mg/kg IV 1200–1500 mg per month	IV infusion, twice per month No titration Infusion center	5.3 days	~ 0.5%	~ 2.5 months
ALZ-801 *Alzheon*	265 mg orally 530 mg daily	Oral tablet, twice daily Titration over 2 weeks Home	36 h	~ 40%	~ 1 week

*Abbreviations*: *IV* intravenous, *SC* subcutaneous

Data sources: [11, 14, 30, 34]

*Assumes adult average weight of 60–75 kg; **time to peak brain steady state concentration (without titration) = 5 times plasma *t*_1/2_ or 5 times dosing frequency, if dosing interval is longer than plasma *t*_1/2_

The key differentiating PK feature of an anti-amyloid agent is the time to achieve steady state concentration in plasma, which allows it to cross the blood-brain barrier and engage and neutralize neurotoxic soluble Aβ oligomers. Due to the relatively long plasma *t*_1/2_ of aducanumab and gantenerumab [11, 34], the time to peak brain levels at steady state, which is required for maximum efficacy, is approximately 5 months. Indeed, with the 6-month titration regimen for aducanumab in phase 3 trials, 10 months of continuous exposure to the highest 10 mg/kg monthly dose was necessary to achieve clinical efficacy [19], which aligns well with the time to achieve sustained peak brain levels. BAN2401 is projected to achieve peak brain exposure in ~ 2.5 months, based on the 10 mg/kg twice monthly dosing selected for the ongoing phase 3 CLARITY AD trial [35]. Considering that no titration period is included in the CLARITY AD trial, the onset of clinical efficacy may occur earlier with BAN2401 than aducanumab.

Higher brain penetration of aducanumab versus BAN2401 does not appear to translate to better efficacy of aducanumab, suggesting that the superior selectivity for oligomers of BAN2401 is a key factor. In addition, the total dose of BAN2401 at the phase 2 and 3 dosing regimens is approximately double that of aducanumab, which may explain some of the efficacy differences. The relatively low brain penetration of anti-amyloid antibodies of less than 1.5% results in low steady state brain concentrations and, when combined with long *t*_1/2_ and dosing intervals, may contribute to variability in pharmacodynamic target engagement and clinical efficacy in individual patients. In contrast, oral ALZ-801 with a shorter plasma *t*_1/2_ and twice daily dosing reaches high steady state peak brain concentrations in ~ 1 week, facilitated by a robust 40% brain penetration [30]. This PK profile of ALZ-801 assures that full inhibition of Aβ oligomer formation is achieved at the target clinical dose, and explains the earlier onset of clinical efficacy in AD patients treated with ALZ-801/tramiprosate compared to antibody treatments [31].

Because the interdose interval of all late-stage antibody treatments is longer than the plasma *t*_1/2_, the peak steady state brain levels are lower than the maximal exposure that could be attained if the interdose interval was equal to or shorter than the *t*_1/2_. The sub-optimal dosing frequency of antibodies further lowers their steady state brain levels and increases the variability in pharmacodynamic target engagement and clinical efficacy, with patients who achieve lower steady state concentrations showing less efficacy.

The time to peak brain levels is an important factor for achieving optimal target engagement and, together with peak brain exposure, drives the clinical benefit. However, even after the peak brain exposure is attained, there is a lag period before the manifestation of maximum clinical benefit, which is dependent on the period of time needed to remove amyloid oligomers or substantially reduce their neurotoxicity [36]. This latency to clinical benefit may be further modified by an individual’s Aβ oligomer burden, APOE4 genotype, age, and disease stage. These factors, when combined with PK variability, can lead to an increase in variability in drug response. Consistent with how these factors interact to impact efficacy, aducanumab’s EMERGE phase 3 results showed the strongest clinical benefit and significance only at the highest dose of 10 mg/kg monthly IV infusion, and only after sufficient number of patients reached sustained peak exposure [19]. The 265 mg dose of ALZ-801 administered twice daily as an oral tablet achieved sustained brain exposures in AD patients that are 5-fold higher than the brain concentration required for full inhibition of formation of neurotoxic oligomers [15, 16]. The sustained CSF drug levels at this dose explain the clinical efficacy observed in APOE4 carriers in the tramiprosate phase 3 trials [24, 31].

For the four late-stage anti-amyloid agents, the rank order for the time to peak exposure and the onset of action is ALZ-801 < < BAN2401 < aducanumab ≈ gantenerumab, as highlighted in Table 3. In summary, PK properties have important implications for the onset of efficacy, time to maximum clinical benefit, and individual variability in clinical response. Given the relatively short time frame available to stabilize the rate of AD decline, and the importance of the perception of efficacy for patient compliance, these PK features are critical for the success of a treatment in a real-world setting.

## Discussion

Major progress has been achieved in AD drug development, with several anti-amyloid agents recently reporting promising clinical and biomarker effects in late-stage trials. The three antibodies aducanumab, gantenerumab, and BAN2401, and the small molecule ALZ-801, have also shown acceptable long-term safety. Therefore, confirmation of their efficacy may lead to the approval and marketing of this first wave of disease-modifying treatments over the next 3 to 5 years.

### Engagement of amyloid oligomers drives clinical and biomarker efficacy

A substantial body of evidence supports the role of Aβ oligomers as early triggers of AD pathology [1–3]. Brain levels of neurotoxic soluble Aβ oligomers, rather than plaques or fibrils, correlate closely with onset and progression of AD symptoms [6, 8, 37]. Aβ oligomers damage synapses, induce tau hyperphosphorylation and neuroinflammation, and impair memory formation in preclinical models, as well as brains of AD patients [8, 37–39]. The most synaptotoxic species are the small oligomers, ranging from dimers to dodecamers, and pyroglutamate forms of Aβ oligomers [40]. Recent clinical trial results with aducanumab, BAN2401, and gantenerumab confirmed that agents that engage amyloid oligomers also reduce downstream tau pathology [13, 19, 20, 22, 23, 27] and cognitive decline [19, 23].

The four late-stage anti-amyloid agents with clinical efficacy and biomarker effects include three injectable antibodies and the oral small molecule, ALZ-801. The common characteristic shared by these agents is their ability to target neurotoxic soluble Aβ oligomers [2, 3]. Aducanumab and gantenerumab preferentially target insoluble amyloid plaques and fibrils and engage oligomers only partially [11, 12], while BAN2401 preferentially targets oligomers over plaques [10], and ALZ-801 selectively blocks the formation of Aβ oligomers without binding amyloid plaque [15–17].

The degree of Aβ oligomer selectivity appears to be a key factor that together with PK properties determines the clinical profile of each anti-amyloid agent. Both aducanumab phase 3 data and BAN2401 phase 2 data showed significant effects on cognition and function [19, 23], and the degree of benefit parallels their respective selectivity for soluble oligomers versus insoluble amyloid plaques (Table 1), with BAN2401 delivering larger clinical benefits likely due to better selectivity for oligomers. Gantenerumab did not show clinical efficacy in the SCarlet RoAD or Marguerite RoAD phase 3 trials [13, 20, 22] and is currently being evaluated in two additional GRADUATE phase 3 trials in early AD to assess the high dose of 1020 mg SC injection administered monthly. Gantenerumab also recently failed on clinical endpoints in the DIAN-TU study in familial AD, potentially due to the small sample size, the mix of presymptomatic and symptomatic subjects, and the late introduction of the higher gantenerumab dose, where only 37% of the subjects received the high dose of 1200 mg monthly SC injections [21]. ALZ-801 which selectively blocks the formation of Aβ oligomers showed significant, clinically meaningful effects on cognition and function in APOE4/4 homozygotes [31], which were similar in magnitude to BAN2401 benefits in APOE4 carriers [23]. One explanation for the higher efficacy of these agents in APOE4 carriers versus noncarriers is their several-fold higher burden of soluble Aβ oligomers [41].

In remarkable contrast to the association between the level of engagement of soluble Aβ oligomers and clinical efficacy, no such association has been reported for the clearance of insoluble amyloid plaques and clinical efficacy. Aducanumab and BAN2401 showed compelling dose-dependent lowering of amyloid plaques in phase 3 and 2 trials [11, 19, 23], with several doses robustly clearing plaque on amyloid PET imaging, yet only long exposures to the highest doses of aducanumab and BAN2401 resulted in significant efficacy, suggesting that prolonged engagement of Aβ oligomers at the highest doses is necessary for clinical benefit. Therefore, plaque clearance alone does not explain clinical efficacy, and amyloid plaque formation may be a protective mechanism by which soluble oligomers are sequestered to limit their neurotoxicity [42]. In line with this hypothesis, the anti-amyloid antibody bapineuzumab and other agents that target primarily amyloid plaques and fibrils failed to show efficacy [43]. Agents that target amyloid monomers, such as the anti-amyloid antibody solanezumab, failed in multiple phase 3 trials [29], or even caused cognitive worsening as observed in phase 3 trials of several beta secretase inhibitors that inhibit the formation of monomers.

All three anti-amyloid antibodies reduced CSF p-tau, the biomarker which best correlates with tau pathology and cognitive decline. While the effective levels of antibodies achieved in the brain and their specificity for amyloid oligomers were predictive of cognitive benefit, this was not the case for CSF p-tau. Although gantenerumab in the DIAN-TU study showed larger p-tau effects than aducanumab and BAN2401 (31% versus 13–15%), it failed to show efficacy on clinical endpoints in the DIAN-TU study or other trials. In addition to CSF p-tau, aducanumab also showed effects on tau PET imaging in the EMERGE trial [19]. Both BAN2401 and gantenerumab phase 2 data showed significant effects on CSF NfL [13, 23, 27], and gantenerumab also showed consistent effects on t-tau. Both NfL and t-tau are considered downstream biomarkers of neuronal injury [44, 45]. The consistency of amyloid antibody effects on CSF p-tau, t-tau, and NfL supports the important role of these biomarkers in future AD trials.

Hippocampal atrophy assessed by volumetric MRI measurements is considered the most reliable structural biomarker of AD progression as noted in the recent draft guidances from both the US Food and Drug Administration (FDA) and European Medicines Agency. Tramiprosate, in the MRI sub-study of a phase 3 trial, showed a dose-dependent decrease in hippocampal atrophy [32], providing the only evidence to date of a significant drug effect on preventing brain atrophy. The effects of ALZ-801 on fluid biomarkers and volumetric MRI measures will be evaluated in a phase 2 study in APOE4 carriers with early AD, as well as a phase 3 trial in APOE4/4 homozygotes with early AD.

The consistency of clinical and biomarker effects of these four anti-amyloid agents provides robust evidence for the role of Aβ oligomers as upstream initiator of AD pathogenesis that triggers tau pathology, neurodegeneration, and resultant cognitive impairment (Table 1). The recent development of sensitive plasma assays for p-tau and NfL applied across the clinical AD continuum provides additional support for the early, initiating role of Aβ in AD pathogenesis and will further facilitate the use of fluid biomarkers in clinical trials [46–48].

### Differences in oligomer selectivity and pharmacokinetic properties determine safety profiles

The selectivity for Aβ oligomers and the individual PK properties underlie the different safety profiles of the anti-amyloid agents, namely the risk of brain edema, ARIA-E, and microhemorrhage, ARIA-H (Tables 1 and 2). The risk of ARIA-E is especially important in APOE4 carriers who carry a higher burden of vascular amyloid. The high ARIA-E rates of aducanumab (35%) and gantenerumab (28–42%), and the lower rate of BAN2401 (10%) in the overall population, parallel their affinity for amyloid plaque binding, and the degree of selectivity for soluble oligomers versus insoluble plaques. ALZ-801/tramiprosate, which does not bind amyloid plaque, has not been associated with events of ARIA-E. The other common adverse events reported with these agents include headache in 20% of patients treated with aducanumab, infusion-related reactions leading to discontinuations in 2.5% of patients treated with BAN2401, and injection site erythema reported in 13% of patients treated with gantenerumab. In the tramiprosate trials, mild to moderate nausea was reported in 20% of patients and vomiting occurred in 14% of patients. The incidence and severity of nausea and vomiting were reduced ~ 50% by the optimized ALZ-801 formulation. In addition to the convenience of oral dosing, the absence of ARIA-E highlights the value of selective oral small molecules that can inhibit the formation or block the effects of Aβ oligomers, without causing activation of immune-mediated plaque clearance and brain edema.

### Benefit-risk profiles of anti-amyloid agents in APOE4 carriers

APOE4 carriers, who are at higher risk of early AD progression and constitute ~ 65% of AD patients, provide an optimal group for clinical efficacy studies and initial approval for drugs targeting neurotoxic soluble Aβ oligomers, since these patients carry a several-fold higher burden of Aβ oligomers compared to noncarriers [41]. APOE4 carriers also have higher levels of insoluble amyloid deposits in brain vessels, which increases their risk of ARIA-E and ARIA-H when treated with anti-amyloid antibodies [33]. Therefore, careful evaluation of efficacy and safety of anti-amyloid agents in APOE4 carriers is important to determine the benefit-risk profile in this large subgroup of AD patients with increased risk and earlier onset of the disease. Due to the rapid removal of amyloid plaque deposits from brain vessels, treatment with antibodies aducanumab, gantenerumab, and BAN2401 is associated with a substantial risk of ARIA-E in APOE4 carriers. While the efficacy of aducanumab in APOE4 carriers has not yet been disclosed, the incidence of ARIA-E in APOE4 carriers was ~ 42% in this group versus ~ 35% in the overall population [11, 19]. BAN2401 showed more robust clinical efficacy in APOE4 carriers [23]; however, this was associated with a higher incidence of ARIA-E of ~ 15% versus 10% in the overall study population. In APOE4/4 homozygotes, ALZ-801/tramiprosate showed substantial cognitive and functional benefits with no events of ARIA-E [31] and may offer an optimal benefit-risk profile in APOE4 carriers.

### The next generation of selective anti-oligomer agents

The next generation of anti-oligomer therapeutics with improved selectivity and product profiles includes the following agents and mechanisms: (1) PMN310, an anti-amyloid antibody that selectively clears formed Aβ oligomers; (2) CT1812, a small molecule that inhibits Aβ oligomer binding to specific neuronal receptors that mediate neurotoxicity; and (3) PQ912 and ALZ-801, small molecules that prevent the formation of neurotoxic soluble Aβ oligomers. An example of an antibody designed to be highly selective to Aβ oligomers is PMN310 from ProMIS Neuroscience [49], which is in preclinical development. CT1812 is an oral small molecule from Cognition Therapeutics that inhibits the binding of Aβ oligomers to sigma-2 receptors [50], which is thought to mediate some of the oligomer-induced synaptic toxicity. CT1812 is currently being tested in phase 2 studies. PQ912 is an oral small molecule inhibitor of glutaminyl cyclase from Vivoryon Therapeutics that inhibits the formation of pyroglutamate forms of Aβ oligomers, which are thought to be highly toxic to synapses. In a small phase 2 study of 12-week duration, PQ912 showed promising effects on a biomarker of neuroinflammation in CSF [51].

ALZ-801 is an oral agent, which efficiently crosses the blood-brain barrier, selectively interacts with Aβ monomers to inhibit misfolding, and blocks the formation of neurotoxic soluble Aβ oligomers in a dose-dependent manner, without affecting insoluble amyloid plaques or fibrils [15–18]. ALZ-801 is a prodrug of tramiprosate with improved gastrointestinal absorption and tolerability, which achieved excellent brain penetration of ~ 40%, with low intersubject variability in over 130 elderly volunteers and AD patients [30]. ALZ-801 is ready to enter phase 3 based on a large body of clinical data in AD patients, including (1) favorable safety profile from tramiprosate safety database of > 2000 AD patients treated for 18 months [24, 31]; (2) well-defined target clinical dose that fully blocks the formation of Aβ oligomers in the brain based on clinical data from the phase 3 AD trials and from an in vitro Aβ oligomer inhibition assay [15, 30]; and (3) precision medicine approach initially focusing on high-risk homozygous APOE4/4 patients with early AD [1]. Oral ALZ-801 provides convenient at-home dosing for elderly patients and their caregivers, as well as potential preventive treatment of presymptomatic subjects with high risk for AD.

## Conclusions

Substantial clinical and biomarker data from late-stage anti-amyloid programs signals a promising new era for AD drug development and provides compelling evidence for the prominent role of neurotoxic soluble amyloid oligomers in the pathogenesis of AD and as therapeutic targets. Considering the pressing need, regulators have shown increasing engagement with drug developers and potential flexibility with respect to conditions and criteria for drug approval. A recent draft guidance from the US FDA on demonstrating substantial evidence of effectiveness [52] seems to allow more flexibility in the evidentiary standards of efficacy. This may pave the way for the approval of these AD drugs in the near future.

This progress also presents an opportunity to build upon the recent data and to develop therapeutics with superior efficacy, safety, and convenience. The benefit-risk profiles of future agents, in particular, the magnitude of clinical benefit and ease of administration, will be key differentiators for patients and their families, physicians, and payers.

## Data Availability

Not applicable

## Abbreviations

Aβ: Beta amyloid
AD: Alzheimer’s disease
ADAS-cog: Alzheimer’s Disease Assessment Scale—cognitive subscale
ADCOMS: Alzheimer’s Disease Composite Score
APOE: Apolipoprotein E
ARIA-E: Amyloid-related imaging abnormalities with effusion or edema
ARIA-H: Amyloid-related imaging abnormalities with hemosiderin deposits
CDR-SB: Clinical Dementia Rating—Sum of Boxes
CSF: Cerebrospinal fluid
DIAN-TU: Dominantly Inherited Alzheimer’s Network Trials Unit
FDA: Food and Drug Administration
IgG: Immunoglobulin G
IV: Intravenous
MOA: Mechanism of action
MRI: Magnetic resonance imaging
NfL: Neurofilament light chain
PET: Positron emission tomography
PK: Pharmacokinetic
p-tau: Phosphorylated tau
SC: Subcutaneous
*t*_1/2_: Half-life
t-tau: Total tau
